# Transforming musculoskeletal injury management using Virtual Bones: a novel digital solution

**DOI:** 10.1136/bmjoq-2025-004121

**Published:** 2026-10-06

**Authors:** Mohamed A Mansour, Ali Al-Kulabi, Minaam Abbas, Layth Qaraqe, Sade Uwaoma, Ahmad Al-Sukaini, Lora Young, Sue Deakin

**Affiliations:** 1 Department of Trauma and Orthopaedic Surgery, West Suffolk Hospital NHS Trust, Bury St Edmund’s, UK; 2 Department of Medicine, University of Cambridge, School of Clinical Medicine, Cambridge, UK; 3 Department of Trauma and Orthopaedic Surgery, Addenbrooke’s Hospital, Cambridge, UK

**Keywords:** Emergency department, Clinical practice guidelines, Continuous quality improvement, Decision support, clinical, Mobile Applications

## Abstract

**Introduction:**

Managing musculoskeletal (MSK) injuries requires effective communication and collaboration between the emergency department (ED) and orthopaedic teams. A quality improvement project analysing virtual fracture clinic (VFC) referrals, staff challenges and the impact of the novel digital solution, Virtual Bones (VB), was undertaken at the West Suffolk Hospital. VB is a digital platform, available at the point of care, that guides clinicians in the assessment and management of MSK injury presentations in ED and referral to VFC.

**Methods:**

VFC outcome data were collected between 2017 and 2022, with a 2-week focused data collection gathering diagnosis, ED processing times, patient leaflet distribution, VFC decisions and referral quality. An ED survey was distributed to explore staff challenges in the management of MSK injuries. Virtual Bones, a novel digital solution, was developed and deployed to enable clinicians to comprehensively digitalise MSK injury management pathways from first patient encounter to referral/discharge. Subsequent data collection cycles compared baseline to post deployment results.

**Results:**

The Virtual Bones pilot (undertaken May–July 2023) involved 352 users, 2400 sessions and 56 700 page views. The initial 2-week cycle included 260 patients while the subsequent cycles included a total of 258. Mean ED processing times decreased from 166 to 122 min, with an up to 90 min reduction in the Foundation Doctors’ group (p<0.0001). Inappropriately referred dischargeable injuries decreased from 23 to 10. Improper documentation decreased from 58% to 30% (p<0.0001). The VFC discharge rate increased from 39% to 48% (p=0.021). Patient information leaflet use increased from 27% to 41% (p=0.032). The ED staff survey demonstrated improved staff confidence in the revealed challenges, including navigating local guidelines, locating equipment, applying immobilisation devices and supplying information leaflets.

**Conclusion:**

Robust engagement with Virtual Bones, along with improved metrics, suggests that this digital solution effectively streamlined MSK injury pathways and helped staff manage challenges.

**WHAT IS ALREADY KNOWN ON THIS TOPIC**

- Musculoskeletal (MSK) injuries place a heavy burden on emergency departments (EDs), where care pathways are often inefficient due to high staff turnover and limited MSK training. Although clinical decision support systems can improve decision-making and efficiency, their use in MSK care remains limited and often non-holistic.

**WHAT THIS STUDY ADDS**

- This study presents and evaluates the Virtual Bones app, a novel, locally tailored digital platform designed to support ED clinicians in the end-to-end management of MSK injuries. Post-implementation data demonstrated statistically significant improvements in ED processing time, referral quality, patient information distribution and reduction in inappropriate VFC referrals. The app was well adopted, with strong user engagement and positive feedback highlighting its usability and practical value in busy clinical environments.

**HOW THIS STUDY MIGHT AFFECT RESEARCH, PRACTICE OR POLICY**

- The Virtual Bones app provides a compelling case for the integration of dynamic, user-informed digital tools to improve adherence to clinical guidelines, reduce system inefficiencies and enhance training for junior staff. Future research will focus on multicentre trials and adaptation across National Health Service trusts.

## Problem

The West Suffolk Hospital (WSH) continues to experience growing demand, with approximately 94 000 emergency department (ED) attendances recorded in 2023–2024.^1^ A significant proportion of these attendances are related to musculoskeletal (MSK) and soft-tissue injuries, which National Health Service (NHS) data report account for 22–39% of all ED attendances.^2^ The MSK injury pathway involves initial assessment, investigation and management by the ED clinician, and often ends with an orthopaedic review, either immediately or at a virtual fracture clinic (VFC).

Several factors within the current pathway may contribute to variation in patient flow through the ED and decision-making within the VFC. Existing Trust guidelines, summarised within an A1 poster covering over 70 injuries, provide brief guidance that can sometimes require additional clarification in clinical practice. Junior resident doctors, many of whom rotate frequently through the department, often have variable prior exposure to MSK injuries and therefore require senior input while developing confidence in MSK management. Challenges in locating and correctly applying splints and casts can also interrupt workflow and require input from more experienced staff. In addition, reliance on printed patient information leaflets, which may be distributed inconsistently during busy periods, can create communication inefficiencies between clinicians and patients. High staff turnover and the use of locum clinicians unfamiliar with local pathways further highlight the importance of easily accessible guidance that supports safe and standardised care. Finally, variability in ED documentation can limit the information available to trauma and orthopaedics (T&O) consultants reviewing cases in the VFC, which may contribute to avoidable face-to-face appointments.

SMART objectives turn vague ideas into clear, actionable plans that are Specific, Measurable, Achievable, Relevant and Time-bound. The primary SMART aim of our project was to support ED practitioners in their assessment, management, documentation and referral of MSK injury-related ED attendances by providing easily accessible tools and trust-specific guidance at the point of care. To show an improvement in this pathway, we quantitatively measured ED processing time, number of inappropriate VFC referrals, documentation quality and discharge rate at VFC. We aimed to demonstrate positive changes in the above measures within 2 months of implementing our digital intervention.

The secondary SMART aim was to overcome staff challenges inherent to ED’s staffing patterns. A questionnaire was distributed to ED staff of various grades about the interpretability of the guidelines, their understanding of immobilisation devices/techniques and locations of key items. We aimed to improve confidence in addressing these challenges within 2 months following our interventions.

## Background

The NHS is under considerable strain, and inefficiency is a key concern. The Lord Darzi Independent Investigation of 2024 identified a concerning drop in the percentage of people seen within 4 hours at A&E Departments, from 94% in 2010 to just over 60% in 2024. Additionally, it was observed that hospital staff numbers have increased by 35%, without a corresponding increase in the number of appointments, operations and procedures, suggesting a fall in productivity due to system inefficiency and logistic challenges.^3^ The Carter Report also identified that improving operational productivity and workflow could save £5 billion per annum.^4^

Trauma and MSK injuries are a significant burden on the NHS and in fact account for 25–39% of patients presenting to the ED according to the NHS A&E Attendances and Emergency Admissions Statistics.^2^ With MSK injuries consistently accounting for the largest proportion of ED attendances, inefficiency in their care pathways compound into a large burden on NHS ED resources.

MSK injuries are numerous and have considerable inter and intra-injury heterogeneity. In the UK, they are managed jointly by the Emergency and Orthopaedic departments. Successful diagnosis and management of MSK injuries heavily rely on the existing skills of the ED clinicians. Clear documentation of the clinical history and clinician’s assessment, as well as conducting the right investigations at the first encounter by the ED clinician, is crucial for a streamlined process. Certain injuries have been deemed safe for discharge without orthopaedic follow-up according to the British Orthopaedic Association (BOA). However, many injuries require orthopaedic review within 72 hours, while others need immediate on-call orthopaedic involvement.^5^ The VFC allows for timely review of cases to meet BOA guidance and reduces the need for face-to-face appointments. An analysis of over 17 000 referrals demonstrates that the introduction of digital referrals (VFC) has enhanced referral quality and improved patient care and flow.^6^ However, not all key details are part of the VFC proforma, and the referral may lack critical information.^7^ Additionally, there may be inappropriate referrals.^8^

We speculated that the inefficiencies highlighted this pathway can be addressed using clinical decision support systems (CDSS). These systems are gaining increased importance, and NHS England has released the 5 Rs and 6 Is in support of these initiatives.^9^ CDSS aim to provide timely best practice information to facilitate decisions on assessment, management and referrals. They can enhance the quality and speed of decision-making, reduce errors and unwarranted variation.

In the case of trauma and MSK injuries, they have been shown to reduce unnecessary scans, improve adherence to guidelines and reduce the length of hospital stay by safely modifying clinician behaviour.^10–13^ There is currently no tool to support ED clinicians holistically in the management of MSK injuries to tackle frontline challenges in the pathway and considerations at VFC.

## Measurement

### Baseline measurements

VFC referral data were retrospectively collected from 1 January 2017 to 31 December 2022 to assess engagement over time. A 2-week detailed review of VFC referrals was conducted by examining referral documents and ED notes. Metrics were chosen to reflect key steps in the management and referral process. The following quantitative metrics were collected:

- Injury details.
- Dischargeable injuries referred to VFC.
- Incomplete VFC referral documentation.
- VFC discharge rate.
- Patient leaflet distribution rate.
- Number of inappropriate referrals.
- Documentations of key findings in referrals.

A digital survey was circulated to ED clinicians through departmental communication channels, including email and staff messaging groups to capture the subjective challenges faced by the ED staff in the management of MSK injuries. Questions asked include

- Whether the guidelines are easy to interpret.
- Whether they have good understanding of immobilisation strategies.
- Whether they can locate and distribute patient information leaflets.
- Whether it is easy to locate the correct splints/casts.
- Do you have any feedback on the MSK injury pathway? (Open-ended feedback)

## Design

A mobile application, made available on iOS and Android, was designed incorporating WSH Trust-specific MSK injury guidance to deliver tailored information to frontline staff directly and instantly. Guidelines used in-app were adapted from the pre-existing guidelines at the Trust, written by ED and T&O consultants in line with national guidelines. The content of the app was reviewed by ED and T&O consultants at the Trust, under the oversight of the departmental clinical leads, to ensure clinical safety and applicability. Developing app content was guided by input from users during a brief testing period where user feedback was collected via an in-app mechanism and used to guide design.

All the injuries described in the hospital guidelines were added to an intuitive, interactive skeleton and injury list. Each injury page was standardised to include key details on documentation and assessment, management and referrals. This is to provide comprehensive guidance for each condition, reduce unnecessary referrals to specialty care and facilitate accurate and rapid management at the first encounter. Pages were supplemented with informative and relevant diagrams, pictures, radiographs and videos to increase user confidence and educate practitioners as they assess MSK injuries (figure 1).

**Figure 1:**
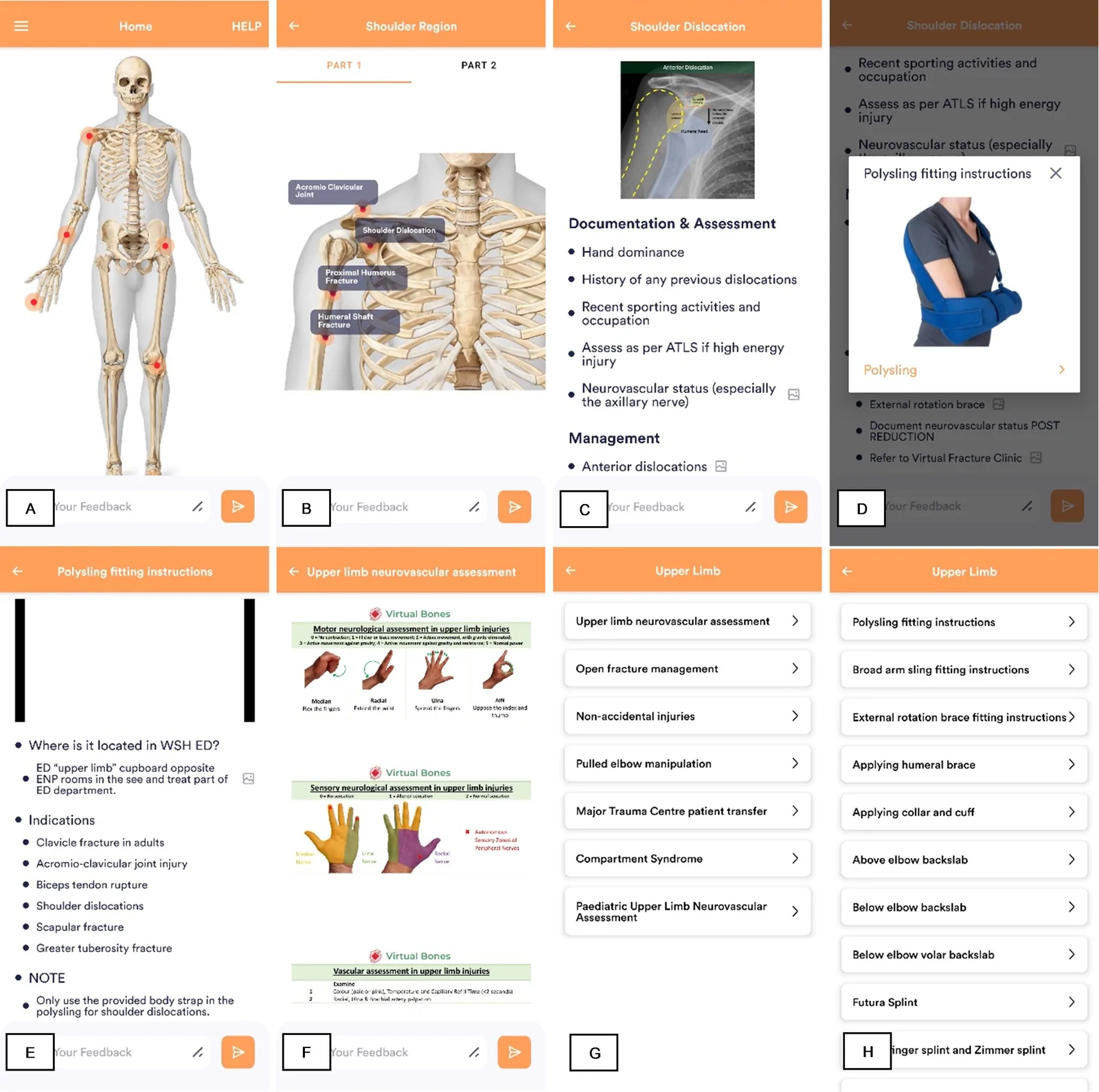
Screens capture from the Virtual Bones mobile application. First row from left to right: (A) homepage, (B) shoulder region part 1, (C) shoulder dislocation injury page and (D) neurovascular assessment. Second row from left to right: (E) polysling fitting instruction pop-out, (F) polysling fitting instruction page, (G) upper limb immobilisation (splints and casts) and (H) upper limb orthopaedic assessments. ENP, Emergency Nurse Practitioner; ATLS, Advanced Trauma Life Support; ED, emergency department; WSH, West Suffolk Hospital.

An orthopaedic assessment section provided didactic support through images and information on the common MSK injury assessments to be conducted at ED. A splint and cast section discussed the use of the immobilisation strategies and displayed videos on their application and their precise locations in the local ED. Both sections are also integrated into each injury page to make the delivery of information seamless and straightforward. To reduce the carbon footprint of MSK injury management and to empower patient understanding and involvement in their own care, the application enabled information leaflets to be sent digitally to patients via email, text message or QR code download. These information leaflets were digital versions of the existing paper leaflets in circulation, which were already approved by ED and T&O consultants at the Trust.

To ensure the application remained dynamic and to accommodate for the newest evidence and practices, a control management system (CMS) in the form of a desktop web application was developed. Nominated admin users can update the application content, including publishing new pathways, guidelines and new locations of splints and casts, with the changes reflecting instantly and push notifications sent to inform users of changes if desired. Push notifications however were not mandatory to avoid clinician alert fatigue.

## Strategy

A PDSA (Plan–Do–Study–Act) cycle is a structured quality improvement method used to test, evaluate and refine changes through iterative intervention. Three PDSA cycles were conducted, with the first lasting 8 months to include app development, and the second and third lasting 1 month each.

### PDSA 1 (September 2022–April 2023)

#### Aim

To evaluate the current MSK injury management and referral pathway, assess ED assessment and documentation for patients referred to VFC, evaluate the efficiency of managing the dischargeable MSK injuries and ascertain the challenges faced by frontline staff at ED in relation to managing MSK injuries.

#### Change strategy and implementation

VFC referrals between 2017 and 2022 were retrospectively explored to reveal the role and referral patterns to VFC. Subsequently, a detailed snapshot of VFC referrals across a 2-week period (26 September 2022 and 9 October 2022) collected additional information including type of injury, ED fracture assessment and documentation, patient leaflet utilisation, VFC outcome and time spent at ED. An ED survey was sent digitally to all ED staff to explore staff challenges.

#### Data and learnings

We found a local 6% annual increase in referrals since 2020. The 2-week snapshot audit showed that some dischargeable injuries were unnecessarily referred to VFC. Furthermore, the majority (60%) of VFC appointments resulted in face-to-face follow-up appointments. Improper documentation by ED clinicians was associated with increased follow-up appointments with an OR of 2. We also identified that leaflets were not provided to patients 73% of the time.

The ED survey revealed the shortcomings of the fracture referral poster, with 8 of 15 respondents finding the poster containing insufficient or unclear information. They recorded the need for additional visual aids and MSK injury teaching to facilitate service provision. An inadequate understanding of the types of immobilisation splints was reported, as was a difficulty pinpointing items and patient leaflets which slowed service provision.

The findings were presented at the local morbidity and mortality meeting on 25 January 2023. It was deemed that a user-friendly, interactive and accessible tool was needed to support staff in the challenges, and to provide accurate and concise guidance in a way that educates ED staff of all grades and experience (including ad hoc locums) on assessing, managing and referring MSK injuries at WSH. Safe adaptability and simple control of this tool was deemed pivotal to ensure it remained dynamic enough to be used to address emerging or recurring problem themes in the MSK injury management pathway at a local level.

### PDSA 2 (May 2023)

#### Aim

To verify the Virtual Bones clinical content with ED and T&O consultants and pilot the Virtual Bones app with frontline ED staff for 1 week. We aimed to collect feedback on functionality, ease of use and examine changes to baseline metrics.

#### Change hypothesis

Based on the literature on clinical decision support tools, it was hypothesised that an improvement in documentation and referral quality would be observed. It was also hypothesised that the enhanced MSK injury guidance available on Virtual Bones would improve decision-making by educating practitioners on how to deal with the frontline challenges.

#### Change strategy and implementation

The VB digital solution was presented at the ED and T&O governance meetings to receive endorsement for use from both clinical teams, as well as approvals from information governance and IT. The clinical content was developed based on the WSH guidelines and verified clinically by T&O and ED consultants directly through VB. The performance metrics were collected and the staff survey administered after this period and compared with baseline. Technical feedback and ease of use was collected through the application’s feedback portal. Pre-launch material such as posters were designed to encourage end-user engagement. Virtual Bones was also included in the ED induction in the form of a presentation and live demonstration. Induction attendees were encouraged to sign up. A departmental email was sent to all ED staff to introduce and encourage further engagement with Virtual Bones.

#### Data and learnings

Usage analytics for Virtual Bones showed 292 users, 1100 sessions and 28 800 page views over a 1-week data collection period post launch. Feedback sent directly via the feedback portal comprised 129 entries allowing local challenges to be expressed by this multidisciplinary staff team. The main feedback themes were to increase radiograph incorporation, add new clinical information and make technological adjustments. Significant improvements in the baseline measures were observed. No clinical or safety incidents were reported by staff or patients following the intervention.

### PDSA 3 (June 2023)

#### Aim

To deploy the latest iteration of Virtual Bones for general use and collect the data for comparison with baseline measurements.

#### Change hypothesis

In line with the previous hypothesis, and since the updates were guided by end users, we hypothesised the latest iteration of Virtual Bones would result in maintained or improved results relative to cycle 2.

#### Change strategy and implementation

Feedback was reviewed by the team and discussed with the relevant consultant specialists prior to implementation. Following approval, all changes were reflected following a system update without disruption to workflow and staffing. A push notification was sent to all users to notify them of updated content. Encouragement of user adoption of the digital solution continued using the change and implementation strategies mentioned in cycle 2.

#### Data and learnings

Usage analytics showed a total of 352 users, 2400 sessions and 56 700 page views. Mild improvements in the baseline measures relative to cycle 2 were observed. Similarly, no clinical or safety incidents were reported by staff or patients.

## Patient and public involvement

Patients and/or the public were not involved in the design, or conduct, or reporting or dissemination plans of this research.

## Results

### Engagement with VFC at WSH

On average, there were 5584 patients referred to the VFC each year with an average of 465 patients each month. Since the beginning of the pandemic in the UK in 2020, referrals to the VFC increased by 6% each year with 6558 referrals made in 2022. This equates to 729 more patients per annum when compared with the number of referrals in 2020.

### Quantitative metrics

The deployment of Virtual Bones and staff engagement with it resulted in improved metrics across all key fields (figure 2A). The initial 2-week data collection involved 260 VFC referrals, while the post VB cycles included a total of 258 (128 in cycle 2 and 130 in cycle 3). The diagnoses making up these referrals remained relatively consistent across cycles, with stable ankle fractures, soft tissue injuries and un-displaced phalangeal fractures being most common (figure 2B). Data from cycles 2 and 3 were combined for statistical analysis. A χ^2^ test was used to assess statistical significance among metrics including improper documentation, patient leaflet distribution and VFC discharge rate. A Student’s t-test was used to evaluate statistical significance in the mean ED processing time. All statistical analyses were performed using Microsoft Excel. Mean ED processing times overall decreased from 166 to 122 min, with up to 90 min reduction in the Foundation Doctors’ group in some cases (p<0.0001). Improper documentation rate decreased from 58% to 30% (p<0.0001). The VFC discharge rate increased from 39% to 48% (p=0.021). Patient information leaflet distribution increased from 27% to 41% (p=0.032). Inappropriately referred dischargeable injuries decreased from 23 to 10 when comparing the 2 weeks prior and post Virtual Bones implementation (figure 3A).

**Figure 2:**
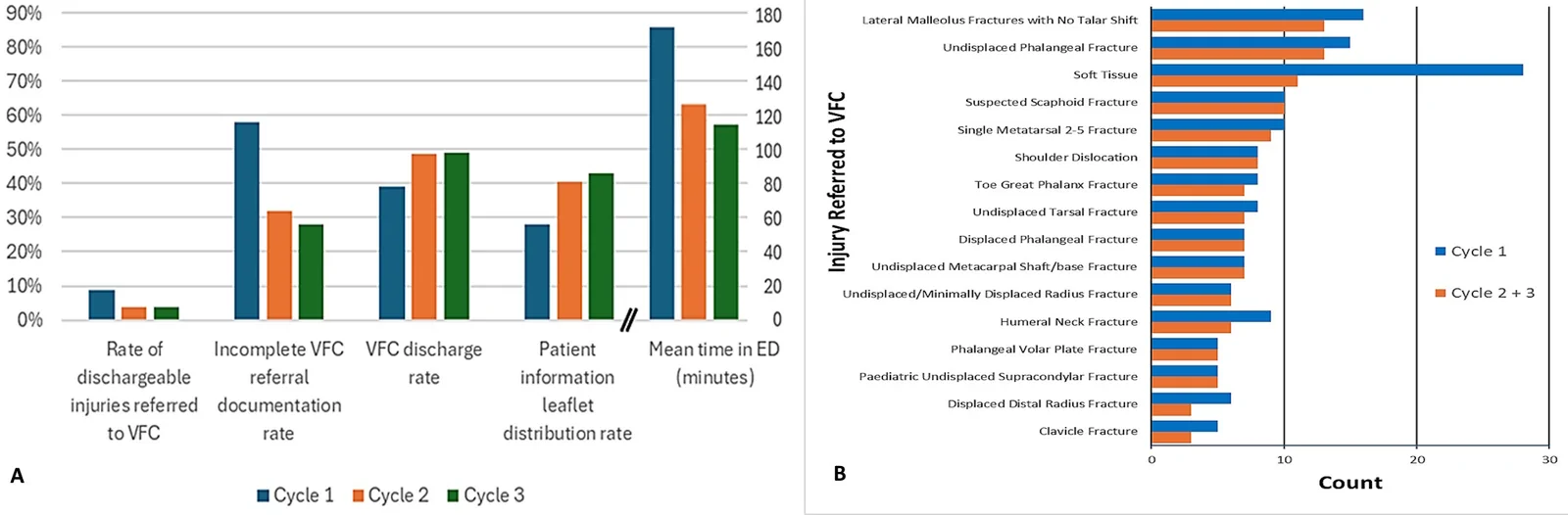
Improved quantitative baseline metrics with consistent virtual fracture clinic (VFC) referral injury frequencies. (A) Change in baseline metrics across cycles. (B) Distribution of the most common presentations in VFC referrals.

**Figure 3:**
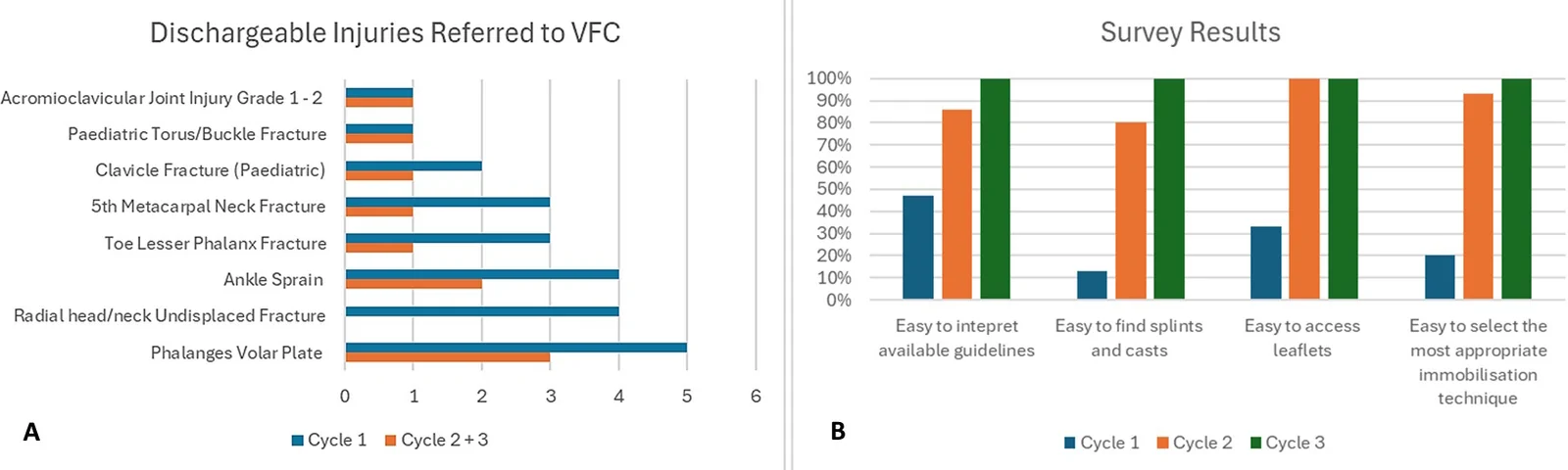
Improved staff confidence with reduced inappropriate virtual fracture clinic (VFC) referrals. (A) Dischargeable injuries referred to VFC. (B) Emergency department staff survey result summary.

### ED staff surveys

The ED staff surveys received 15 responses in cycle 1, 15 responses in cycle 2 and 16 responses in cycle 3. Respondents in cycle 1 comprised four registrars, seven senior house officers and four foundation year doctors. Cycle 2 included five registrars, nine senior house officers and one foundation year doctor, while cycle 3 included four registrars, eight senior house officers and four foundation year doctors. Following the deployment of the latest iteration of Virtual Bones, all respondents scored guideline interpretability, finding and selecting splints/casts accurately and leaflet distribution as easy (figure 3B).

## Lessons and limitations

This project has highlighted the clear benefits of using digital solutions to address common inefficiencies in the MSK injury pathways within emergency care. The development and implementation of the Virtual Bones platform demonstrated that targeted, user-informed digital tools can meaningfully improve staff performance including improving patient processing times, documentation quality, guideline adherence and reducing unnecessary referrals. Both objective and subjective data were collected to better determine evidence of impact.

A key lesson was the value of collaborative co-design and early engagement with both ED and T&O teams. This allowed the intervention to directly respond to local frontline challenges and facilitated end-user engagement. Due to early involvement, resistance to change for this project was also reduced as key stakeholders were already on board. This approach was supported by a systemic review that encouraged active involvement of users.^14^

Another key lesson was the value of dynamic content and continuous feedback. The built-in feedback mechanism and Content Management System (CMS) allowed for rapid responses and actions to user criticism and feedback. This was kept as a core feature as continuous engagement ensured the tool remained useful and evidence based.

Improved ED processing times for patients presenting to ED with an MSK injury were demonstrated. The similarity in type of injuries across each cycle strengthens the findings by minimising confounding from variation in management times between different injuries. The greatest reduction in processing time was seen among Foundation Doctors who have the least experience interpreting radiographs and MSK injury management.^15–17^ Notably, this improvement occurred without additional teaching activities to the Foundation Doctors’ cohorts. No clinical or safety concerns were reported by patients or staff during any PDSA cycle or up to the time of publication, suggesting that the practice changes were implemented safely and that doctors continued to work within the limits of their competence. Staff challenges were well addressed using the Virtual Bones solution, which likely reflects the working cohort’s preference for easy access, simplified, digital information. It also revealed areas that are not commonly included in ED inductions and training.

This project had several limitations. Patient samples in each cycle were identified through VFC referrals, in line with the project’s aims of streamlining both ED management of MSK injuries and the quality of VFC referrals. However, Virtual Bones provides comprehensive guidance for over 70 MSK presentations, including many that do not require VFC referral. While a reduction in avoidable VFC referrals was observed following implementation, the project did not quantitatively assess the tool’s impact for injuries that fall outside the VFC referral pathway.

The evaluation was also limited to a single district general hospital site, potentially restricting generalisability. The data collection periods, spanning 4 weeks in total, offered a short-term snapshot of impact. Larger studies across multiple sites are required to validate this tool’s ability to improve patient management and referrals of MSK injuries. Additionally, the content is tailored to be local-specific, which serves as a strength to the tool at the WSH, but also mandates minor adaptation for other ED departments. Adaptation and tailoring of the contents according to a hospital’s local protocols is estimated to require one site visit and 1–2 weeks of development. Practically, this involves capturing the locations of splints and casts photographically and descriptively, incorporating a Trust’s protocols and approved digital leaflets via the CMS, consultant verification of content and testing the application. Engagement with information governance and information technology teams adds an additional step to implementation of Virtual Bones at new sites that are considering evaluation of the tool. As this work was primarily focused on clinician-facing tools and aimed at addressing operational and referral inefficiencies, patient and public involvement was not sought at this early stage. Nonetheless, patient participation remains a meaningful and valuable next step.

## Conclusion

The Virtual Bones project successfully met its primary and secondary SMART aims by improving the quality, efficiency and consistency of MSK injury management within the ED setting. The intervention addressed key gaps in clinical documentation, decision-making and referral processes, resulting in a marked reduction in inappropriate VFC referrals, increased distribution of patient information leaflets, reduced ED processing times and increased staff confidence.

This project adds to existing evidence on the valuable role of digital decision support tools in enhancing frontline clinical practice and uniquely contributes an example of a locally adaptable, interactive and holistic digital solution that addresses both operational workflow challenges as well as educational needs within a high-turnover workforce. Multi-site evaluation is an important next step in determining the effectiveness of this tool and the digitalisation approach to MSK injury guidance in general.

## Data Availability

Data are available upon reasonable request.
